# Supplementary figures and images for: Articulated remains of the extinct shark Ptychodus (Elasmobranchii, Ptychodontidae) from the Upper Cretaceous of Spain provide insights into gigantism, growth rate and life history of ptychodontid sharks
Source: PLoS One. 2020 Apr 22;15(4):e0231544. doi: 10.1371/journal.pone.0231544 (PMC7176087; doi:10.1371/journal.pone.0231544)

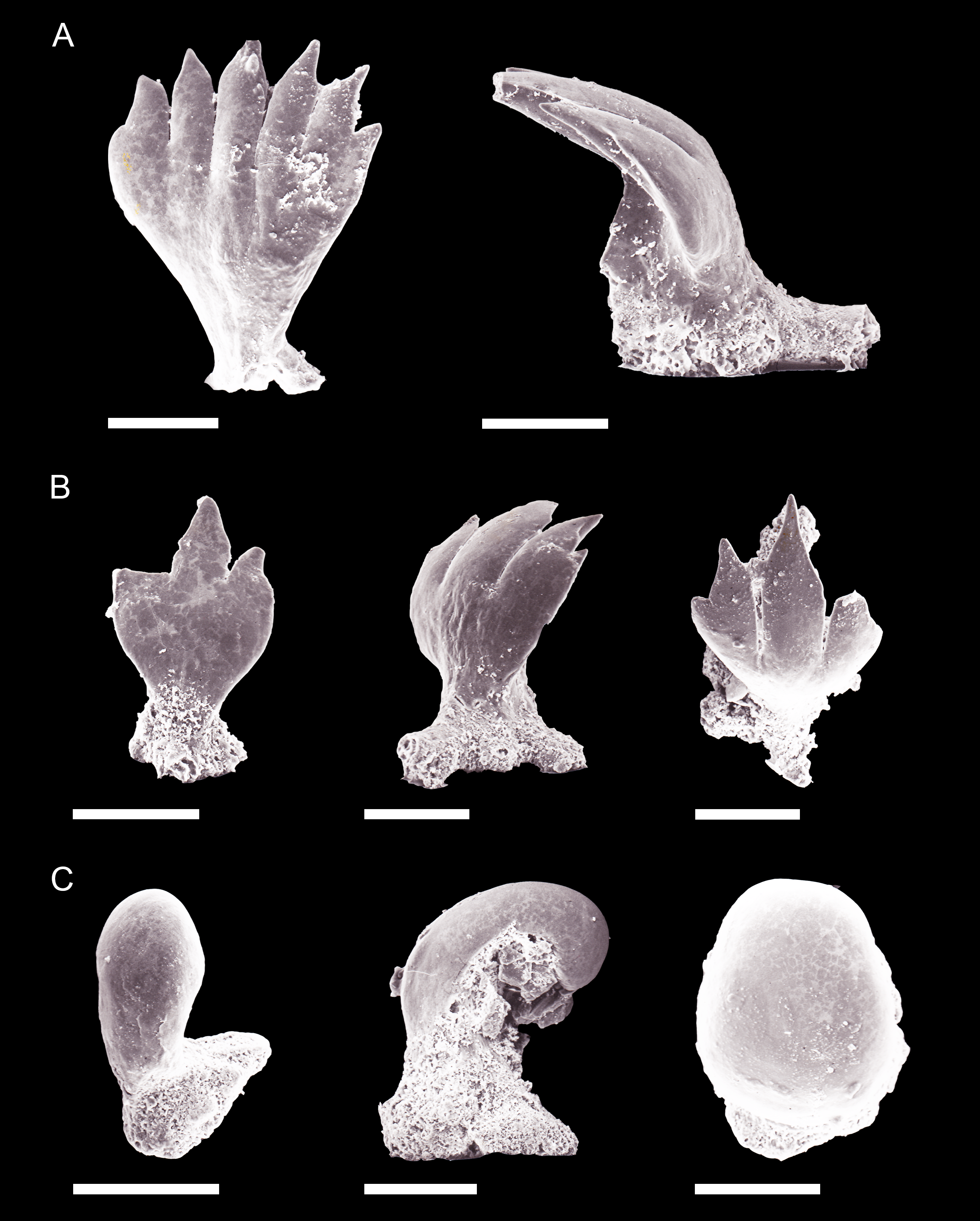

Supplement: S1 Fig — (A) type 1, “six-keeled scales”; (B) type 2, “three-keeled scales”; (C) type 3, “knob-like scales”. Scale bar equals 100μm. (TIF) [file pone.0231544.s001.tif]

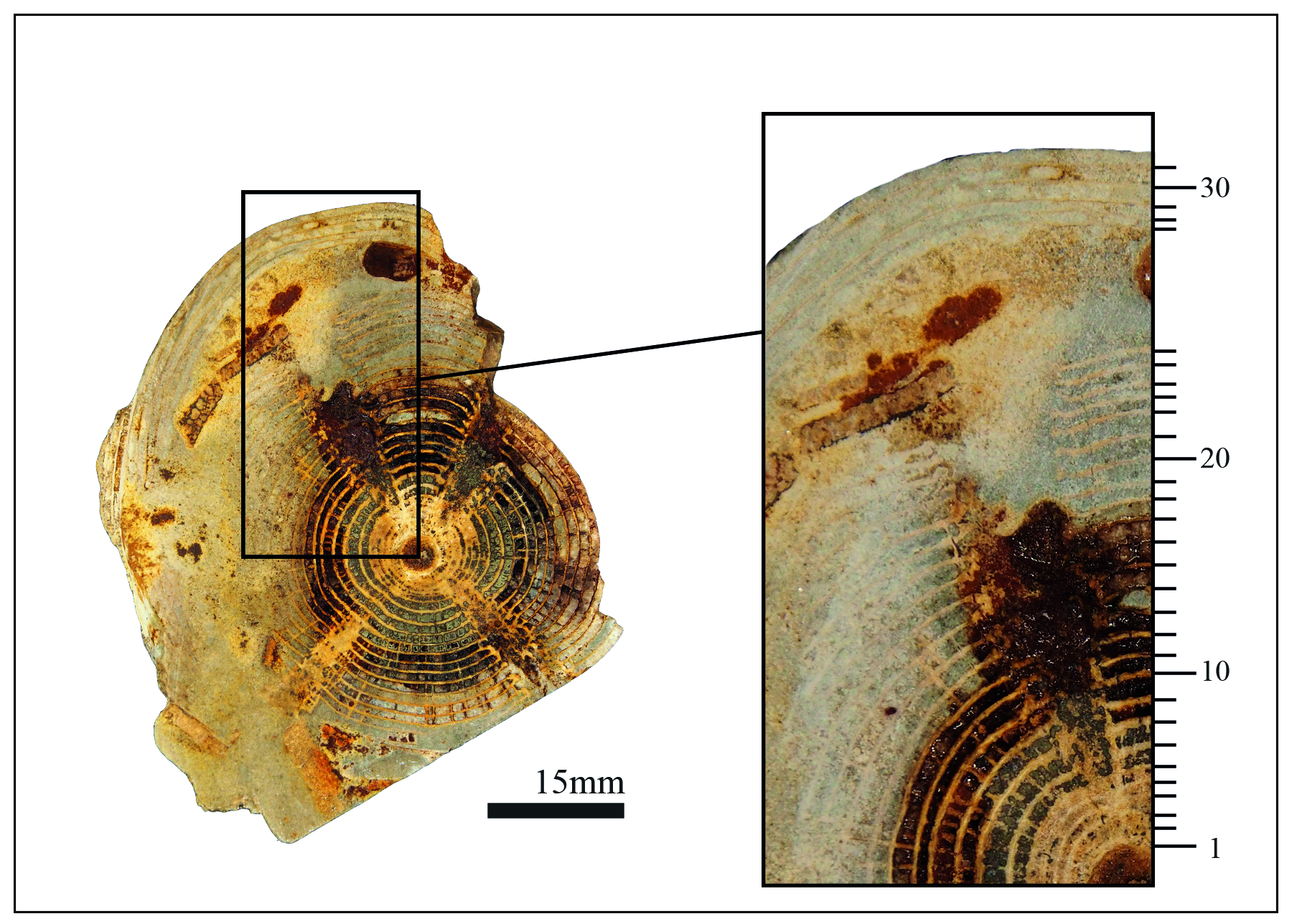

Supplement: S2 Fig — (TIF) [file pone.0231544.s002.tif]
