# Supplementary material for: Articulated remains of the extinct shark Ptychodus (Elasmobranchii, Ptychodontidae) from the Upper Cretaceous of Spain provide insights into gigantism, growth rate and life history of ptychodontid sharks
Source: PLoS One. 2020 Apr 22;15(4):e0231544. doi: 10.1371/journal.pone.0231544 (PMC7176087; doi:10.1371/journal.pone.0231544)
Supplement: Attachment — Submitted filename: PONE-D-20-05473_reviewer JA.pdf [file pone.0231544.s003.pdf]

PLOS ONE

First articulated remains of the extinct shark, Ptychodus (Elasmobranchii, Ptychodontidae) from the Upper Cretaceous of Spain provide insights into gigantism, growth rate and life history of ptychodontid sharks

--Manuscript Draft--

**Manuscript Number:**	PONE-D-20-05473
**Article Type:**	Research Article
**Full Title:**	First articulated remains of the extinct shark, Ptychodus (Elasmobranchii, Ptychodontidae) from the Upper Cretaceous of Spain provide insights into gigantism, growth rate and life history of ptychodontid sharks
**Short Title:**	Life history of the giant Cretaceous shark Ptychodus
**Corresponding Author:**	Patrick L. Jambura, MSc<br>Universitat Wien<br>Wien, AUSTRIA
**Keywords:**	Vertebrate paleontology; Cretaceous; sharks; paleoecology; life history; vertebrae; Elasmobranchii; Chondrichthyes
**Abstract:**	Due to their cartilaginous endoskeleton and the continuous tooth replacement, the chondrichthyan fossil record predominantly consists of isolated teeth, which offer diagnostic features for taxonomic identifications, but only provide very limited information of an organism's life history. In contrast, the calcified vertebral centra of elasmobranchs (sharks, skates and rays) yield important information about ecological and biological traits that can be utilized for constructing age-structured population dynamic models of extant species and palaeoecological reconstructions of such aspects in extinct groups. Here, we describe two large shark vertebrae from the Santonian (Upper Cretaceous) of Spain, which show a unique combination of characters (asterospondylic calcification pattern, with concentric lamellae and numerous parallel bands that are oriented perpendicular) that is only known from ptychodontid sharks, a distinct, extinct group of giant durophagous sharks of the Cretaceous era. Based on linear regression models for large galeomorph sharks a total length between 430 and 707cm was estimated for the examined specimen. Our results indicate that ptychodontid sharks were large viviparous animals, with slow growth rates, matured very late and, therefore, show typical traits for K-selected species. These traits combined with a highly specialized feeding ecology might have played a crucial role for this group's success but also, eventually, extinction.
**Order of Authors:**	Patrick L. Jambura, MSc
	Jürgen Kriwet
**Additional Information:**	
**Question**	**Response**
**Financial Disclosure**<br><br>Enter a financial disclosure statement that describes the sources of funding for the work included in this submission. Review the submission guidelines for detailed requirements. View published research articles from *PLOS ONE* for specific examples.<br><br>This statement is required for submission and **will appear in the published article** if the submission is accepted. Please make	The authors received no specific funding for this work.<br>Open access funding provided by University of Vienna.

sure it is accurate.<br><br>**Unfunded studies**<br>Enter: *The author(s) received no specific funding for this work.*<br><br>**Funded studies**<br>Enter a statement with the following details:<br>• Initials of the authors who received each award<br>• Grant numbers awarded to each author<br>• The full name of each funder<br>• URL of each funder website<br>• Did the sponsors or funders play any role in the study design, data collection and analysis, decision to publish, or preparation of the manuscript?<br>• **NO** - Include this sentence at the end of your statement: *The funders had no role in study design, data collection and analysis, decision to publish, or preparation of the manuscript.*<br>• **YES** - Specify the role(s) played.<br><br>* typeset	
**Competing Interests**<br><br>Use the instructions below to enter a competing interest statement for this submission. On behalf of all authors, disclose any competing interests that could be perceived to bias this work—acknowledging all financial support and any other relevant financial or non-financial competing interests.<br><br>This statement **will appear in the published article** if the submission is accepted. Please make sure it is accurate. View published research articles from *PLOS ONE* for specific examples.	The authors have declared that no competing interests exist.

**NO authors have competing interests**<br><br>Enter: *The authors have declared that no competing interests exist.*<br><br>**Authors with competing interests**<br><br>Enter competing interest details beginning with this statement:<br><br>*I have read the journal's policy and the authors of this manuscript have the following competing interests: [insert competing interests here]*<br><br>* typeset	
**Ethics Statement**<br><br>Enter an ethics statement for this submission. This statement is required if the study involved:<br><br>• Human participants<br>• Human specimens or tissue<br>• Vertebrate animals or cephalopods<br>• Vertebrate embryos or tissues<br>• Field research<br><br>Write "N/A" if the submission does not require an ethics statement.<br><br>General guidance is provided below. Consult the submission guidelines for detailed instructions. **Make sure that all information entered here is included in the Methods section of the manuscript.**	N/A

**Format for specific study types**<br><br>**Human Subject Research (involving human participants and/or tissue)**<br>• Give the name of the institutional review board or ethics committee that approved the study<br>• Include the approval number and/or a statement indicating approval of this research<br>• Indicate the form of consent obtained (written/oral) or the reason that consent was not obtained (e.g. the data were analyzed anonymously)<br><br>**Animal Research (involving vertebrate animals, embryos or tissues)**<br>• Provide the name of the Institutional Animal Care and Use Committee (IACUC) or other relevant ethics board that reviewed the study protocol, and indicate whether they approved this research or granted a formal waiver of ethical approval<br>• Include an approval number if one was obtained<br>• If the study involved *non-human primates*, add *additional details* about animal welfare and steps taken to ameliorate suffering<br>• If anesthesia, euthanasia, or any kind of animal sacrifice is part of the study, include briefly which substances and/or methods were applied<br><br>**Field Research**<br><br>Include the following details if this study involves the collection of plant, animal, or other materials from a natural setting:<br>• Field permit number<br>• Name of the institution or relevant body that granted permission	
**Data Availability**<br><br>Authors are required to make all data underlying the findings described fully available, without restriction, and from the time of publication. PLOS allows rare exceptions to address legal and ethical concerns. See the PLOS Data Policy and FAQ for detailed information.	Yes - all data are fully available without restriction

A Data Availability Statement describing where the data can be found is required at submission. Your answers to this question constitute the Data Availability Statement and **will be published in the article**, if accepted.<br><br>**Important:** Stating 'data available on request from the author' is not sufficient. If your data are only available upon request, select 'No' for the first question and explain your exceptional situation in the text box.<br><br>Do the authors confirm that all data underlying the findings described in their manuscript are fully available without restriction?	
**Describe where the data may be found in full sentences. If you are copying our sample text, replace any instances of XXX with the appropriate details.**<br><br>• If the data are **held or will be held in a public repository**, include URLs, accession numbers or DOIs. If this information will only be available after acceptance, indicate this by ticking the box below. For example: *All XXX files are available from the XXX database (accession number(s) XXX, XXX.).*<br>• If the data are all contained **within the manuscript and/or Supporting Information files**, enter the following: *All relevant data are within the manuscript and its Supporting Information files.*<br>• If neither of these applies but you are able to provide **details of access elsewhere**, with or without limitations, please do so. For example:<br><br>*Data cannot be shared publicly because of [XXX]. Data are available from the XXX Institutional Data Access / Ethics Committee (contact via XXX) for researchers who meet the criteria for access to confidential data.*<br><br>*The data underlying the results presented in the study are available from (include the name of the third party*	All relevant data are within the manuscript and its Supporting Information files.

and contact information or URL).<br>• This text is appropriate if the data are owned by a third party and authors do not have permission to share the data.<br><br>* typeset	
Additional data availability information:	

Manuscript Click here to access/download;Manuscript;Manuscript_final.docx

First articulated remains of the extinct shark, *Ptychodus* (Elasmobranchii, Ptychodontidae) from the Upper Cretaceous of Spain provide insights into gigantism, growth rate and life history of ptychodontid sharks

Author names: Patrick L. Jambura[1*], Jürgen Kriwet[1]

Addresses: [1]University of Vienna, Department of Palaeontology, Vienna, Austria

Short Title: Life history of the giant Cretaceous shark *Ptychodus*

*Corresponding author

Email: patrick.jambura@gmail.com (PLJ)

Abstract

Due to their cartilaginous endoskeleton and the continuous tooth replacement, the chondrichthyan fossil record predominantly consists of isolated teeth, which offer diagnostic features for taxonomic identifications, but only provide very limited information of an organism's life history. In contrast, the calcified vertebral centra of elasmobranchs (sharks, skates and rays) yield important information about ecological and biological traits that can be utilized for constructing age-structured population dynamic models of extant species and palaeoecological reconstructions of such aspects in extinct groups. Here, we describe two large shark vertebrae from the Santonian (Upper Cretaceous) of Spain, which show a unique combination of characters (asterospondylic calcification pattern, with concentric lamellae and numerous parallel bands that are oriented perpendicular) that is only known from ptychodontid sharks, a distinct, extinct group of giant durophagous sharks of the Cretaceous era. Based on linear regression models for large galeomorph sharks a total length between 430 and 707cm was estimated for the examined specimen. Our results indicate that ptychodontid sharks were large viviparous animals, with slow growth rates, matured very late and, therefore, show typical traits for K-selected species. These traits combined with a highly specialized feeding ecology might have played a crucial role for this group's success but also, eventually, extinction.

Introduction

More than 400 million years of evolution have shaped a diverse set of biological traits in modern elasmobranchs (sharks, skates and rays) that allowed them to occupy a variety of different niches and trophic levels. They have conquered marine and freshwater environments around the world, reaching body sizes from 0.2m (dwarf lantern shark *Etmopterus perryi*) to 20m (whale shark *Rhincodon typus*), and have developed a number of different reproduction strategies that can be roughly categorized in oviparity (egg laying), and viviparity (giving birth to live pups) [1,2]. Three general life history patterns can be found in sharks: 1) small body size, low longevity, small litters, small offspring, fast growth; 2) large body size, moderate to high longevity, large litters, small offspring, small growth; 3) large body size, high longevity, small litters, large offspring, slow growth [3].

The application of life history traits has proven potentially useful in fisheries to determine if stocks are endangered and to estimate their chances to recover [4,5]. Additionally, life history traits of extinct taxa are vital for palaeoecological reconstructions and can give important insights into a species' demise, perseverance, and are crucial to augment our understanding of diversity and extinction patterns [6-8]. However, the fossil record of chondrichthyans predominantly consists of isolated teeth, which only offer limited information about a species' biological traits. Vertebrae on the other hand yield important data on the biology and ecology of fossil elasmobranchs, but only have been described for a very limited number of extinct species [9-14].

Ptychodontid sharks seemingly were giant durophagous fish that lived in the Cretaceous period from the Aptian (~113-125mya) to the Campanian (~72-83mya) [15] and are believed to have obtained body sizes of more than ten meters [16,17]. Although fossils of this group are

common in Cretaceous deposits and are known from around the globe, the taxonomic placement of this group remains ambiguous and they have been discussed to be either batomorphs [18], hybodont sharks [16], or put in the new order Ptychodontiformes within the Neoselachii (sensu Compagno [19]; Elasmobranchii sensu Maisey [20]) [21]. However, the presence of calcified vertebrae [22] and a three-layered enameloid [23] support the affinity of this group to modern sharks.

Here we describe the first articulated shark remains from the Santonian of Spain, Europe. Although no teeth were found associated with the vertebrae, taxonomic placement was possible due to a unique combination of characters that is only known for ptychodontids and allowed the exclusion of any other shark taxon known from this period. Furthermore, the vertebrae yield important information about the ontogeny, growth and body size and, therefore, provide insights into the life history of this enigmatic shark group.

Material and methods

Geographic and geological setting

The material that forms the focus of this study comprises a portion of an axial skeleton consisting of five articulated and several disarticulated vertebral centra belonging to the same individual, which were collected 10 km west of Santander in northern Spain, from a section on the coast near the village of Soto de la Marina (Fig 1). Here, sediments ranging from the Cenomanian to Maastrichtian accumulating to 1200 m in thickness are well exposed. The investigated section starts with light greyish to whitish, massive and arenitic limestone beds forming the lower unit reaching a thickness of 24.7m. This lower unit ranges from the late Campanian to early Santonian in age and is characterized by the occurrence of the holasteroid, *Cardiaster integer*

(‘*integer* limestones’), which is a well-known species from the calcareous platforms of the Basque-Cantabrian Region, northern and southern Pyrenees, and Alpes-Maritimes [24].

Within the ‘*integer* limestones’, two layers characterized by abundant occurrences of the inoceramid, *Platyoceramus* (*Cladoceramus*) *undulatoplicatus* were identified during fieldwork for a diploma thesis by K. Oppermann (Berlin, Germany) in 1997 [25] and designated as *undulatoplicatus* events I and II. The first appearance of *Platyceramus undulatoplicatus* marks the base of the Santonian stage according to the Santonian Working Group report to the second Symposium on Cretaceous Stage Boundaries [26]. The vertebrae, which were recovered from a ca. 1.2 x 0.5m large, concretionary limestone lens coming from the upper, 2nd *undulatoplicatus* Event, consequently are of earliest Santonian age.

Fig 1. Fossil locality near Santander, northern Spain where EMRG-Chond-SK-1 was recovered (indicated by a black star).

Documentation and preparation

The articulated vertebral section was photographically documented in the field but not collected (Fig 2). Small sediment samples adjacent to the incomplete vertebral column were taken for screen-washing and two disarticulated vertebral centra of varying preservational degree, which are housed in the fossil vertebrate collection of the Department of Palaeontology (University of Vienna) under the number EMRG-Chond-SK-1. Of one vertebral centrum, a dorso-ventrally directed thin section was prepared. The sediment samples and carbonate matrix of the other specimens were removed with 10% acetic acid and the residues sieved with a 0.25 mm mesh and

sorted under a binocular. The recovered material comprises some dermal scales of the elasmobranch placoid type, which are deposited in the fossil collection of the Institute of Palaeontology, Free University Berlin without numbers. Some of these placoid scales were studied with a Jeol high-vacuum scanning electron microscope at the Institute of Geological Sciences of the Free University Berlin, Germany (S1 Fig).

Fig 2. Additional articulated (A, B) and disarticulated shark vertebrae (C,D) found in situ associated with EMRG-Chond-SK-1. Picture courtesy of K. Oppermann, 1997.

Size estimation

Previous studies showed a linear relationship between total length (TL) and vertebral centrum diameter (CD) in several shark species [27-31] that can be expressed with a regression equation [10,32]. Due to the fragmentary nature of the material, we measured the radius and multiplied it by two to obtain the diameter of the centrum. The larger vertebra EMRG-Chond-1b had a sedimentary infill between increment rings 25 and 26. Taking this into account, the radius was measured to the 25th increment ring and the distance between the 26th and outermost increment ring (31) was added. The distance between band pair 25 and 26 was estimated as the average value of the band intervals of the five preceding and five succeeding increment rings. As it was impossible to determine the position of the two isolated vertebrae, we conservatively regarded the vertebra with the higher centrum diameter as the largest vertebra in the entire individual. This

approach ensured a minimum estimate for the total length and reduced the risk of overestimating the body size.

Most pelagic sharks have a consistent body form [10,33], therefore, it was regarded as reasonable in previous studies to assume that the relationship between vertebral size and total body length is consistent between species with similar ecologies. However, it has been reported that this relationship can vary between species [9,10,12]. To overcome this issue we conducted two independent approaches to estimate the total length of the examined specimen:

(1) We extrapolated the total length of EMRG-Chond-SK-1b by comparing it with the estimated total length (TL) and centrum diameter (CD) of †*Ptychodus occidentalis* [16].

$$TLx(cm) = TL_{\dagger\ Ptychodus\ occidentalis} \times \frac{CD_{EMRG-Chond-SK-1b}}{CD_{\dagger\ Ptychodus\ occidentalis}}$$

(2) We used published regression equations for large galeomorph sharks to estimate the relationship between centrum diameter (CD; mm) and total length (TL; cm). The following species with known regression equations were used as templates: (1) the great white shark *Carcharodon carcharias* [32]; (2) tiger shark *Galeocerdo cuvier* [34]; (3) whale shark *Rhincodon typus* [31].

$$TL_{Carcharodon\ carcharias} = 22 + 5.8 \times CD$$

$$TL_{Galeocerdo\ cuvier} = 35.293 + 14.314 \times CD$$

$$TL_{Rhincodon\ typus} = 36.695 + 9.531 \times CD$$

Results and Discussion

Systematic Palaeontology

Class CHONDRICHTHYES Huxley, 1880 [35]

Subclass ELASMOBRANCHII Bonaparte, 1838 [36]

Order *incertae sedis*

Family PTYCHODONTIDAE Jaekel, 1898 [18]

Genus *PTYCHODUS* Agassiz, 1835 [37]

Material. EMRG-Chond-SK-1; two vertebral centra.

Locality. Soto de la Marina, west of Santander, Cantabria, N Spain.

Age and horizon. Early Santonian, 'integer limestone', *undulatoplicatus* Events II.

Description. Both centra were found articulated with several other vertebrae, which were not recovered and left in the field (Fig 2). Vertebra centra show a marked concavity on the articular surfaces (amphicoelous) and appear nearly circular in median transverse view. The dimensions are approximately 55mm (EMRG-Chond-SK-1a) and 70mm (EMRG-Chond-SK-1b) in diameter (dorsoventrally) and the vertebral anterior-posterior length is 23mm (EMRG-Chond-SK-1a). Due to the fragmentary condition of the material, no dimensions could be measured for the mediolateral diameter.

Anterior and posterior facets of the vertebral centra show concentric calcareous rings extending outwards from the center of the vertebrae. Numerous parallel lamellae are oriented perpendicular to these concentric lamellae (Fig 3A). EMRG-Chond-SK-1b was sectioned transversely and exhibits an asterospondylic calcification pattern (Fig 3B): secondary calcification leaves four uncalcified areas (i.e. two basidorsal and two ventral cartilage wedges), which radiate diagonally from the center to the base of the neural and haemal arches. Concentric lamellae are restricted to the intermedialia (the area between the wedges). A total of 31 growth increments can be identified in the vertebra section. Postmortem sedimentary infilling can be

observed between ring 25 and 26 which inflated the distance between those two rings (Fig 3B). The dorsoventral and mediolateral radii of the first increment ring (birth ring) are 3.6 and 3.2mm, respectively.

Fig 3. Morphology and calcification pattern of the vertebral centra of EMRG-Chond-SK-1. (A) close up view and illustration of the outer morphology of EMRG-Chond-SK-1a; (B) vertebral section in transverse plane and illustration of EMRG-Chond-SK-1b.

Taxonomic remarks

In contrast to teeth, vertebral centra are thought to bear only little taxonomic information for extinct elasmobranch fishes as comparative analyses are hard to perform due to the lack of articulated material with associated teeth. Hasse [38] recognized three different calcification patterns of vertebrae in cross-section (cyclospondyl, tectospondyl, and asterospondyl), which later was revised by Ridewood [39], who stated that these three categories were not sufficient to describe the plethora of different calcification patterns that can be found in sharks, skates and rays. The vertebral centrum EMRG-Chond-SK-1b displays the asterospondylic type (sensu Hasse [38]) with four uncalcified areas radiating from the center to the bases of the neural and haemal arches, which is typical for galeomorph sharks [40].

Another character that is apparent in the cross section is the presence of concentric lamellae that are extending outwards from the centra. The combination of these two features (asterospondyly with uncalcified wedges and concentric lamellae) are only known from the

basking shark *Cetorhinus maximus* [39,41], the whale shark *Rhincodon typus* [28], †*Ptychodus* [42,43], and †*Squalicorax* [43,44]. Both †*Ptychodus* and †*Squalicorax* are known from Cretaceous deposits in Europe, N- and S-America, Africa and Asia [15]. Our specimen shows numerous parallel bands that are oriented 360° around the center of the vertebrae. These parallel lamellae are oriented perpendicular to the concentric lamellae, a trait only known from ptychodontid sharks and is regarded as a diagnostic feature for this group, which is absent in other sharks, including *C. maximus*, *R. typus*, and †*Squalicorax* [21,45]. This assumption is also supported by previous reports of ptychodontid shark vertebrae, which display this feature [22,42,46]. Rozefelds [47] reported large vertebral centra from the lower Cretaceous Toolebuc Formation of Australia, which resemble our specimens. He found associated placoid scales but no oral teeth and assigned the species tentatively to the anacoracid genus †*Pseudocorax*. ~~This, however,~~ seems very unlikely because †*Pseudocorax* was a rather small anacoracid shark with tooth crown heights of 1.5cm [15], which is comparable to the teeth of a two meters long †*Squalicorax falcatus* [9]. Furthermore, like in our specimen, parallel lamellae vertebrae are visible, which is not known from anacoracid sharks. The combination of the above mentioned characters (parallel lamellae, concentric lamellae, asterospondyl centra), the size of the vertebral centra and the stratigraphic age (Cretaceous) of these species, leads us to the assumption that both specimens, EMRG-Chond-SK-1 and Rozefelds' QMF18264, are ptychodontid sharks. Other big sharks from the Late Cretaceous are known from the order Lamniformes (e.g., †*Cretalamna*, †*Cretodus*, †*Cretoxyrhina*), but can easily be ruled out because they are known to have a different mineralization pattern of the vertebral centra (i.e., the vertebral centrum is strengthened by multi-branched, densely packed lamellae), have radial lamellae on the dorsoventral axis in lateral view (which our specimen does not have), and lack parallel lamellae

vertebrae [12,13,44,48-51]. The placoid scales from the sediment samples associated with the Spanish specimen represent three different morphotypes (see Supporting Information S1 Fig). Placoid scale type 3 of the Spanish specimen resembles those figured by Shimada et al. [16,17] and the 'curved crown scale' type of Rozefelds [47] to some extent. The other two scale types in the specimen described here have not been figured before. These morphological differences might be related to different positions of the scales on the body. As no teeth were found associated with this specimen, an exact taxonomic identification on species level remains impossible, which leaves us to refer to it as †*Ptychodus* sp.

Age, growth and inferred life history traits

Age estimation

Whole vertebral centra, as well as transverse and sagittally sectioned centra have been commonly used for aging elasmobranchs by counting alternating opaque and translucent band pairs (also referred to as band pairs, annuli, rings, or vertebral growth increments) that are deposited in the vertebral centra as they grow [10,21,52-54]. A number of studies suggested that these band pairs are deposited annually in several elasmobranch species e.g., in the shortfin mako shark *Isurus oxyrinchus* [29], scalloped hammerhead shark *Sphyrna lewini* [55], dusky shark *Carcharhinus obscurus* [56], leopard shark *Triakis semifasciata* [57], smalltooth sawfish *Pristis pectinata* [58], etc. [59-66]. However, this is questioned by recent studies [67-69] and especially in old individuals of long living species the age determination seems to be highly underestimated as the growth rate decreases with age resulting in a band width decline at the centrum edges that can become unresolvable in older individuals [52,67,70-74]. The section of EMRG-Chond-SK-1b revealed 31 band pairs (birth mark + 30 band pairs), suggesting, under the assumption of an

annual growth band deposition, that EMRG-Chond-SK-1 was around 30 years old. These band pairs were well distinguishable, indicating that EMRG-Chond-SK-1 had not reached the maximum length when it died, because no compressed arrangement of band pairs is preserved at the edges.

Body Size Estimation

Based on the previously published centrum diameter and estimated total length of †*Ptychodus occidentalis* [16] we calculated an estimated total length of 8.87-11.83m for EMRG-Chond-SK-1. However, this estimation should be taken with caution, as the TL-CD relationship of †*P. occidentalis* is based on a single vertebral centra which not necessarily represents the largest vertebrae in this specimen and, therefore, ~~bears a great risk to be erroneous and to~~ result in overestimated size approximations. Therefore, we recommend taking the previously estimated TL of 13m for †*P. rugosus* [16], which was also based on this TL-CD relationship, with much caution.

Further indication of overestimated body sizes for †*Ptychodus* is given by our calculations of the total length of EMRG-Chond-SK-1 based on regression equations for large galeomorph sharks. In contrast to the above-mentioned extrapolation, this approach has the advantage, by assuming EMRG-Chond-SK-1b to be the largest vertebrae, to offer minimum size estimations and, therefore, reducing the risk of overestimating the size. Using the vertebral diameter of 70 mm and the regression equations for the great white shark *Carcharodon carcharias* [32], tiger shark *Galeocerdo cuvier* [34] and whale shark *Rhincodon typus* [31], the minimum total length of EMRG-Chond-SK-1 is calculated to be 4.30m, 5.39m, and 7.07m respectively. The use of regression equations for three different species (from three different orders) has shown significant variations in estimated body sizes and, thus, indicates that previous

assumptions of the more or less consistent body forms in pelagic sharks resulting in similar size estimations [33] were oversimplified. In fact, a variety of different factors (e.g., phylogenetic affiliations, lifestyle, trophic level, etc.) might contribute to the relationship between vertebral diameter and total body length and, therefore, affect size estimations. However, it seems reasonable to assume that the total length of EMRG-Chond-SK-1 lies within the estimated range of 4.3-7.07m as the three template species of the regression equations cover a wide range of different ecologies (microphagous and macrophagous sharks) and are not closely related to each other (diversification of these three groups occurred in the Early and Middle Jurassic, respectively [8]). Given that our specimen most likely has not yet reached maturity and therefore represents a subadult, previous size estimations of around 10m [17] seem possible for this group. Although more accurate maximum size estimations need to wait until a complete specimen can be analyzed, our study agrees with previous work that †*Ptychodus* was one of the largest durophagous vertebrates in Earth's history.

Growth Rate

The intervals between adjacent band pairs remain more or less stable from the innermost band pair to the outermost band pair, although some variability does exist (Table 1). This differs from previous studies [10-13], which reported the intervals to decrease from the innermost to the outermost band pairs and indicates that the growth rate of EMRG-Chond-SK1 has not decreased until its death (Fig 4A). Attempts to fit the data to a von Bertalanffy growth model resulted in erroneous maximum size estimations (TL_{max}>20m). Plotting the CR of each growth ring against the growth ring count ("age") resulted in a graph that followed a linear model which, because of the linearity between CR/CD and TL, follows the same trend if TL is plotted against growth ring

count (or “age”) (Fig 4B). Most growth models for fish are nonlinear [75] and shark growth models are usually best described by an asymptotic growth model (e.g., von Bertalanffy growth model) [54]. To date, all examined sharks and rays show asymptotic growth during ontogeny and it therefore seems justified to assume that †*Ptychodus* did so as well. Our data suggest that EMRG-Chond-SK1 has not reached the plateau of the asymptote of the growth curve yet and, consequently, not its maximum length. Furthermore, clear inflections of the growth curve, that indicate a decreased growth rate (e.g., after reaching maturity when energy from somatic growth is diverted to gonadal growth), are absent in EMRG-Chond-SK1 and, thus, suggests that it has not reached maturity at band pair 25 with an estimated body size between 369 and 607cm. These estimations are reasonable when compared to modern giant sharks (“gigantism” sensu Pimiento *et al.* [76] refers to sharks with body sizes exceeding six meters), which show similar sizes at maturity, e.g., the great white shark, *Carcharodon carcharias*, at 350-500cm (TL about 600cm), basking shark, *Cetorhinus maximus*, at 400-800cm (TL more than 1000cm), whale shark, *Rhincodon typus*, at 600-800cm (TL 1700-2100cm) [2]. When compared to big macropredatory sharks (i.e., great white shark *Carcharodon carcharias* and *Cretoxyrhina mantelli*), it is apparent that the slope of the growth curve of †*Ptychodus* is less steep and more similar to the microphagous basking shark *Cetorhinus maximus* (Fig 4C). Therefore, the growth rate of †*Ptychodus* is assumed to be lower than those of apex predatory sharks. Under the assumption of an annual growth band deposition, †*Ptychodus* matured very late (after more than 25 years) and showed great longevity, similar to today's giant filter-feeding sharks [31,77].

Table 1. Measurements and derived estimations taken from vertebra EMRG-Chond-SK-1b.

BN	CR (mm)	BI (mm)	pCD (%)	TL$_{CC}$ (cm)	TL$_{GC}$ (cm)	TL$_{RT}$ (cm)
1	3.7	-	10.51%	64.92	88.25	107.23
2	4.4	0.7	12.50%	73.04	98.28	120.57
3	5.3	0.9	15.06%	83.48	111.16	137.72
4	6.1	0.8	17.33%	92.76	122.61	152.97
5	6.9	0.8	19.60%	102.04	134.06	168.22
6	8.1	1.2	23.01%	115.96	151.24	191.10
7	9.5	1.4	26.99%	132.20	171.28	217.79
8	10.5	1	29.83%	143.80	185.59	236.85
9	12	1.5	34.09%	161.20	207.06	265.44
10	12.9	0.9	36.65%	171.64	219.94	282.60
11	14.2	1.3	40.34%	186.72	238.55	307.38
12	15.6	1.4	44.32%	202.96	258.59	334.06
13	16.8	1.2	47.73%	216.88	275.77	356.94
14	18	1.2	51.14%	230.8	292.95	379.81
15	19.3	1.3	54.83%	245.88	311.55	404.59
16	20.4	1.1	57.95%	258.64	327.30	425.56
17	21.4	1	60.80%	270.24	341.61	444.62
18	22.5	1.1	63.92%	283.00	357.36	465.59
19	24.1	1.6	68.47%	301.56	380.26	496.09
20	25.3	1.2	71.88%	315.48	397.44	518.97
21	26.4	1.1	75.00%	328.24	413.18	539.94
22	27.2	0.8	77.27%	337.52	424.63	555.19
23	28.1	0.9	79.83%	347.96	437.52	572.34
24	29	0.9	82.39%	358.40	450.40	589.50
25	29.9	0.9	84.94%	368.84	463.28	606.65
26	N/A	N/A	N/A	N/A	N/A	N/A
27	N/A	0.8	N/A	N/A	N/A	N/A
28	N/A	0.7	N/A	N/A	N/A	N/A
29	N/A	1.1	N/A	N/A	N/A	N/A
30	N/A	1.1	N/A	N/A	N/A	N/A
31	*35.2	0.8	100.00%	430.32	539.15	707.68

Abbreviations: BN, band number; **CR**, centrum radius; **BI**, band interval; **pCD**, percent centrum diameter from the center of the vertebra **TL**$_{CC}$, total length estimation based on the regression equation of *Carcharodon carcharias* [32]; **TL**$_{GC}$, total length estimation based on the regression equation of *Galeocerdo cuvier* [34]; **TL**$_{RT}$,total length estimation based on the regression equation of *Rhincodon typus* [31].
*reconstructed value; see "Materials and methods" for more details on its computation.

Fig 4. Growth ring profile of EMRG-Chond-SK-1b: (A) centrum size profiles of EMRG-Chond-SK-1b follow the same trend for radii in dorsal, ventral and mediolateral direction. (B) data points of the centrum size profile closely follow a linear function. (C) growth ring distances of EMRG-Chond-SK-1b compared to †*Cretoxyrhina mantelli* [10] and *Cetorhinus maximus* [77].

Inferred life history

Vertebral centra of sharks have proven useful for estimating body sizes [13,33], growth rates [10-13,29,30,64] and, therefore, allow the reconstruction of biological and ecological traits, as various of these aspects of an organism are correlated with body size [78-81]. Although we could not determine the identity of EMRG-Chond-SK-1 on species level, it gives us valuable insights into the ontogeny and ecology of ptychodontid sharks. Our analysis on the vertebral growth indicates that †*Ptychodus* was slow growing, late maturing and seemingly long-living, all of which are key traits for a K-selected species. Based on the radius of the birth ring (0.37cm) we estimated a total length of 0.65-1.07m for our specimen at the time of birth. Offspring with similar sizes are known from a variety of large viviparous sharks, e.g. great hammerhead shark *Sphyrna mokarran* (50-70cm), tiger shark *Galeocerdo cuvier* (51-76cm), great white shark *Carcharodon carcharias* (110-160cm), basking shark *Cetorhinus maximus* (150-170cm), and whale shark *Rhincodon typus* (55-64cm) [2]. Similar sizes at birth have not been reported for oviparous sharks (usually not exceeding 15-25cm), which leads us to the assumption that †*Ptychodus* also was a viviparous shark that put a lot of resources into the development of large offspring.

To date, we can only speculate about the reasons for the extinction of this group. However, K-selected life history strategies represent specific adaptations (slow growing, late maturity, large body, small size of litter) that make such sharks more prone to changing environments and have been correlated with increased extinction risk compared to oviparous (r-selected) sharks [82]. We unambiguously demonstrate here that extinct ptychodontid sharks had

K-selected traits, which in combination with a highly specialized trophic niche (durophagy) might have been a major intrinsic contributor to this group's demise.

Acknowledgements

We are deeply indebted to Kurt Oppermann (Berlin, Germany) for the possibility to study the vertebral centra and placoid scales of this interesting extinct shark, and for providing geographic and geological information. We are grateful to Manuel Amadori and Sebastian Stumpf (both from the University of Vienna, Austria) for discussions and to Kenshu Shimada for providing pictures of an articulated skeleton of *Squalicorax falcatus* which comprises 86 vertebrae. Open access funding provided by University of Vienna.

References

1. Compagno LJV. Alternative life-history styles of cartilaginous fishes in time and space. Environ Biol Fishes. 1990;28: 33-75.
2. Ebert DA, Fowler S, Compagno LJV. Sharks of the World: A Fully Illustrated Guide. Plymouth: Wild Nature Press; 2013.
3. Cortés E. Life history patterns and correlations in sharks. Rev Fish Sci. 2000;8: 299-344.
4. Reynolds JD, Jennings S, Dulvy NK. Life histories of fishes and population response to exploitation. In: Reynolds JD, Mace GM, Redford KH, Robinson JG, editors. Conservation of exploited species. Cambridge: Cambridge University Press; 2001. pp. 147-168.

5. Cortés E. Perspectives on the intrinsic rate of population growth. Methods Ecol Evol. 2016;7: 1136-1145. doi: 10.1111/2041-210X.12592

6. Purvis A, Jones KE, Mace GM. Extinction. BioEssays. 2000;22: 1123-1133. doi: 10.1002/1521-1878(200012)22%3A12<1123%3A%3AAID-BIES10>3.0.CO%3B2-C

7. Cardillo M, Mace GM, Jones KE, Bielby J, Bininda-Emonds ORP, Sechrest W, Orme CDL, Purvis A. Multiple causes of high extinction risk in large mammal species. Science. 2005;309: 1239-1241. doi: 10.1126/science.1116030

8. Kriwet J, Kiessling W, Klug S. Diversification trajectories and evolutionary life-history traits in early sharks and batoids. Proc R Soc B. 2009;276: 945-951. doi: 10.1098/rspb.2008.1441

9. Shimada K, Cicimurri DJ. Skeletal anatomy of the Late Cretaceous shark, *Squalicorax* (Neoselachii: Anacoracidae). Palaeontol Z. 2005;79: 241-261. doi: 10.1007/BF02990187

10. Shimada K. Ontogenetic parameters and life history strategies of the Cretaceous lamniform shark, *Cretoxyrhina mantelli*, based on the vertebral growth increments. J Vertebr Paleontol. 2008;28: 21-33.

11. Cook TD, Newbrey MG, Murray AM, Wilson MVH. Shimada K, Takeuchi GT, Stewart JD. A partial skeleton of the Late Cretaceous lamniform shark, *Archaeolamna kopingensis*, from the Pierre Shale of Western Kansas, U.S.A. J Vertebr Paleontol. 2011;31: 8-21. doi: 10.1080/02724634.2011.539968

12. Newbrey MG, Siversson M, Cook TD, Fotheringham AM, Sanchez RL. Vertebral morphology, dentition, age, growth, and ecology of the large lamniform shark *Cardabiodon ricki*. Acta Palaeontol Pol. 2015;60: 877-897. doi: 10.4202/app.2012.0047

13. Shimada K, Everhart MJ. A new large Late Cretaceous lamniform shark from North America, with comments on the taxonomy, paleoecology, and evolution of the genus *Cretodus*. J Vertebr Paleontol. 2019; e1673399. doi: 10.1080/02724634.2019.1673399

14. Kriwet J, Mewis H, Hampe O. A partial skeleton of a new lamniform mackerel shark from the Miocene of Europe. Acta Palaeontol Pol. 2015;60: 857-875. doi: 10.4202/app.00066.2014.

15. Cappetta H. Handbook of Paleoichthyology, Vol. 3E: Chondrichthyes Mesozoic and Cenozoic Elasmobranchii: Teeth. München: Verlag Dr Friedrich Pfeil; 2012.

16. Shimada K, Rigsby CK, Kim SH. Partial skull of Late Cretaceous durophagous shark, *Ptychodus occidentalis* (Elasmobranchii: Ptychodontidae), from Nebraska, U.S.A. J Vertebr Paleontol. 2009;29: 336-349. doi: 10.1671/039.029.0226

17. Shimada K, Everhart MJ, Decker R, Decker PD. A new skeletal remain of thr durophagous shark, *Ptychodus mortoni*, from the Upper Cretaceous of North America: an indication of gigantic body size. Cretac Res. 2010;31: 249-254. doi: 10.1016/j.cretres.2009.11.005

18. Jaekel, O. 1898. Ueber die verschiedenen Rochentypen. Sitzungsberichte der Gesellschaft naturforschender Freunde zu Berlin 1898:44-53.

19. Compagno LJV. Phyletic relationships of living sharks and rays. Am Zool. 1977;17: 303-322.

20. Maisey JG. What is an ‘elasmobranch’? The impact of palaeontology in understanding elasmobranch phylogeny and evolution. J Fish Biol. 2012;80: 918-951. doi: 10.1111/j.1095-8649.2012.03245.x

21. Hamm SA. First associated tooth set of *Ptychodus anonymous* (Elasmobranchii, Ptychodontidae) in North America from the Jetmore Chalk in Kansas. Trans Kans Acad Sci. 2019;122: 1-18. doi: 10.1660/062.122.0101

22. Everhart MJ, Caggiano T. An associated dentition and calcified vertebral centra of the Late Cretaceous elasmobranch, *Ptychodus anonymus* Williston 1900. Paludicola. 2004;4: 125-136.

23. Hoffman BL, Hageman SA, Claycomb GD. Scanning electron microscope examination of the dental enameloid of the Cretaceous durophagous shark *Ptychodus* supports neoselachian classification. J Paleontol. 2016;90: 741-762. doi: 10.1017/jpa.2016.64

24. Gallemí J, Küchler T, Lamolda M, López G, Martínez R, Muñoz J, Pons JM, Soler M, 1997. The Coniacian-Santonian boundary in north-ern Spain: the Olazagutia section. Min Slovaca. 1997;29: 311.

25. Kriwet J, Oppermann K. First articulated shark remains (Neoselachii, Lamniformes, Anacoracidae) from the Late Cretaceous of Spain. J Vert Paleontol, Abstracts of Papers. 1997:58A.

26. Lamolda MA, Hancock JM. 1996. The Santonian Stage and substages. In: Rawson PF, Dhondt AV, Hancock JM, Kennedy WJ, editors. Proceedings "Second International Symposium on Cretaceous Stage Boundaries" Brussels 8-16 September, 1995. Bull Inst Roy Sci Nat Belgique, Sci Terre; 1996. 66. pp. 95-102.

27. Stevens JD. Vertebral rings as a means of age determination in the blue shark (*Prionace glauca* L.). J mar biol Ass UK. 1975;55: 657-665.

28. Wintner SP. Preliminary study of vertebral growth rings in the whale shark, *Rhincodon typus*, from the east coast of South Africa. Environ Biol Fishes. 2000;59: 441-451.

29. Ribot-Carballal MC, Galván-Magaña F, Quiñónez-Velàzquez C. Age and growth of the shortfin mako shark, *Isurus oxyrinchus*, from the western coast of Baja California Sur, Mexico. Fish Res. 2005;76: 14-21.

30. Tanaka S, Kitamura T, Mochizuki T, Kofuji K. Age, growth and genetic status of the white shark (*Carcharodon carcharias*) from Kashima-nada, Japan. Mar Freshw Res. 2011;62: 548-556.

31. Hsu HH, Joung SJ, Hueter RE, Liu KM. Age and growth of the whale shark (*Rhincodon typus*) in the north-western Pacific. Mar Freshw Res. 2014;65: 1145-1154. doi: 10.1071/MF13330

32. Gottfried MD, Compagno LJV, Bowman SC. Size and skeletal anatomy of the giant megatooth shark *Carcharodon megalodon*. In: Klimley AP, Ainley DG, editors. Great White Sharks: The Biology of *Carcharodon carcharias*. San Diego: Academic Press; 1996. pp. 55-89.

33. Frederickson JA, Schaefer SN, Doucette-Frederickson JA. A gigantic shark from the Lower Cretaceous Duck Creek Formation of Texas. PLoS ONE. 2015;10: e0127162. doi: 10.1371/journal.pone.0127162.

34. Holmes BJ, Peddemors VM, Gutteridge AN, Geraghty PT, Chan RWK, Tibbetts IR, Bennett MB. Age and growth of the tiger shark *Galeocerdo cuvier* off the east coast of Australia. J Fish Biol. 2015;87: 422–448. doi: 10.1111/jfb.12732

35. Huxley TH. On the application of the laws of evolution to the arrangement of the Vertebrata, and more particularly of the Mammalia. Proc Zool Soc Lond. 1880;1880: 649–661.

36. Bonaparte CL. Synopsis vertebratorum systematis. Nuovi Ann Sci nat Bologna. 1838;2: 105–133.
37. Agassiz L. Recherches sur les poissons fossiles. Neuchatel: Petitpierre; 1833–1843.
38. Hasse C. Das Natürliche System der Elasmobranchier auf Grundlage des Baues und der Entwicklung ihrer Wirbelsäule. Eine Morphologische und Paläontologische Studie. Allgemeiner Theil. Jena: Gustav Fischer; 1879.
39. Ridewood WG. On the calcification of the vertebral centra in sharks and rays. Philos Trans R Soc Lond. 1921;210: 311–407.
40. Cappetta H. Handbook of Paleoichthyology, Vol. 3B: Chondrichthyes II Mesozoic and Cenozoic Elasmobranchii. Schultze HP, editor. Stuttgart: Gustav Fischer Verlag; 1987.
41. Natanson LJ, Wintner SP, Johansson F, Piercy A, Campbell P, De Maddalena A, Gulak SJB, Human B, Cigala Fulgosi F, Ebert DA, Hemida F, Mollen FH, Vanni S, Burgess GH, Compagno LJV, Wedderburn-Maxwell A. Ontogenetic vertebral growth patterns in the basking shark *Cetorhinus maximus*. Mar Ecol Prog Ser. 2008;361: 267-278. doi: 10.3354/meps07399
42. Woodward AS. The Fossil Fishes of the English Chalk. Part VI. Palaeontogr Soc Monogr. 1911;64: 185–224.
43. Welton BJ, Farish RF. The Collector's Guide to Fossil Sharks and Rays from the Cretaceous of Texas. Lewisville: Before Time; 1993.
44. Applegate SP. The vertebrate fauna of the Selma Formation of Alabama. Part VIII. The fishes. Chicago: Field Museum of Natural History; 1970.
45. Hamm SA. The Late Cretaceous shark, *Ptychodus rugosus*, (Ptychodontidae) in the Western Interior Sea. Trans Kans Acad Sci. 2010;113: 44-55. doi: 10.1660/062.113.0203

46. Amadori M, Amalfitano J, Giusberti L, Fornaciari E, Carnevale G, Kriwet J. A revision of the Upper Cretaceous shark *Ptychodus mediterraneus* Canavari, 1916 from northeastern Italy, with a reassessment of *P. latissimus and P. polygyrus* Agassiz, 1835 (Chondrichthyes; Elasmobranchii). Cretac Res. 2020; doi: 10.1016/j.cretres.2020.104386
47. Rozefelds AC. Lower Cretaceous Anacoracidae? (Lamniformes: Neoselachii); vertebrae and associated dermal scales from Australia. Alcheringa. 1993;17: 199-210.
48. Shimada K. Skeletal anatomy of the Late Cretaceous lamniform shark, *Cretoxyrhina mantelli*, from the Niobrara Chalk in Kansas. J Vertebr Paleontol. 1997;17: 642-652.
49. Shimada K. Skeletal and dental anatomy of lamniform shark, *Cretalamna appendiculata*, from Upper Cretaceous Niobrara Chalk of Kansas. J Vertebr Paleontol. 2007;27: 584-602.
50. Amalfitano J, Giusberti L, Fornaciari E, Dalla Vecchia FM, Luciani V, Kriwet J, Carnevale G. Large deadfalls of the ‘ginsu’ shark *Cretoxyrhina mantelli* (Agassiz, 1835) (Neoselachii, Lamniformes) from the Upper Cretaceous of northeastern Italy. Cretac Res. 2019;98: 250-275. doi: 10.1016/j.cretres.2019.02.003
51. Conte GL, Fanti F, Trevisani E, Guaschi P, Barbieri R, Bazzi M. Reassessment of a large lamniform shark from the Upper Cretaceous (Santonian) of Italy. Cretac Res. 2019;99: 156-168. doi: 10.1016/j.cretres.2019.02.011
52. Cailliet GM, Martin LK, Kusher D, Wolf P, Welden BA. Techniques for enhancing vertebral bands in age estimation of California elasmobranchs. NOAA tech rep NMFS. 1983;8: 157-165.

53. Cailliet GM, Goldman KF. Age determination and validation on chondrichthyan fishes. In: Carrier JC, Musick JA, Heithaus MR, editors. Biology of sharks and their relatives. Boca Raton: CRC Press; 2004. pp. 399-447.

54. Cailliet GM, Smith WD, Mollet HF, Goldman KJ. Age and growth studies of chondrichthyan fishes: the need for consistency in terminology, verification, validation, and growth function fitting. Environ Biol Fishes. 2006;77: 211-228. doi: 10.1007/s10641-006-9105-5

55. Kotas JE, Mastrochirico V, Petrere Jr M. Age and growth of the Scalloped Hammerhead shark, *Sphyrna lewini* (Griffith and Smith, 1834), from the southern Brazilian coast. Braz J Biol. 2011;71: 755-761. doi: 10.1590/S1519-69842011000400022.

56. Joung SJ, Chen JH, Chin CP, Liu KM. Age and Growth of the Dusky Shark, *Carcharhinus obscurus*, in the Western North Pacific Ocean. Terr Atmos Ocean Sci. 2015;26: 253-160. doi: 10.3319/TAO.2014.10.15.01(Oc)

57. Smith SE. Timing of vertebral-band deposition in tetracycline-injected leopard sharks. Trans Am Fish Soc. 1984;113: 308-313.

58. Scharer RM, Patterson Ill WF, Carlson JK, Poulakis GR. Age and growth of endangered smalltooth sawfish (*Pristis pectinata*) verified with LA-ICP-MS analysis of vertebrae. PLoS ONE. 2012;7: e47850. doi: 10.1371/journal.pone.0047850

59. Branstetter S, McEachran JD. Age and growth of four Carcharhinid sharks common to the Gulf of Mexico: A summary paper. In: Uyeno T, Arai R, Taniuchi T, Matsuura K, editors. Proceedings of the second international conference on Indo-Pacific fishes. Tokyo: Ichthyological Society of Japan; 1986. pp. 361-371.

60. Conrath LC, Gelsleichter J, Musick JA. Age and growth of the smooth dogfish (*Mustelus canis*) in the northwest Atlantic Ocean. Fish Bull. 2002;100: 674-682.

61. MacFadden BJ, Labs-Hochstein J, Quitmyer I, Jones DS. Incremental growth and diagenesis of skeletal parts of the lamnoid shark *Otodus obliquus* from the early Eocene (Ypresian) of Morocco. Palaeogeogr Palaeoclimatol Palaeoecol. 2004;206: 179-192. doi: 10.1016/j.palaeo.2004.01.002

62. Joung SJ, Chen CT, Lee HH, Liu HH. Age, growth, and reproduction of silky sharks, *Carcharhinus falciformis*, in northeastern Taiwan waters. Fish Res. 2008;90: 78-85. doi: 10.1016/j.fishres.2007.09.025

63. Booth AJ, Foulis AJ, Smale MJ. Age validation, growth, mortality, and demographic modeling of spotted gully shark (*Triakis megalopterus*) from the southeast coast of South Africa. Fish Bull. 2011;109: 101-112.

64. Barreto R, Lessa RP, Hazin FH, Santana FM. Age and growth of the blacknose shark, *Carcharhinus acronotus* (Poey, 1860) off the northeastern Brazilian Coast. Fish Res. 2011;110: 170-176. doi: 10.1016/j.fishres.2011.04.003.

65. Enajjar S, Bradai MN, Bouain A. Age, growth and sexual maturity of the blackchin guitarfish *Rhinobatos cemiculus* in the Gulf of Gabès (southern Tunisia, central Mediterranean). Cah Biol Mar. 2012;53: 17-23.

66. Hall NG, Bartron C, White WT, Dharmadi, Potter IC. Biology of the silky shark *Carcharhinus falciformis* (Carcharhinidae) in the eastern Indian Ocean, including an approach to estimating age when timing of parturition is not well defined. J Fish Biol. 2012;80: 1320-1341. doi: 10.1111/j.1095-8649.2012.03240.x

67. Harry AV. Evidence for systematic age underestimation in shark and ray ageing studies. FISH FISH. 2018;19: 185-200. doi: 10.1111/faf.12243

68. Natanson LJ, Skomal GB, Hoffmann SL, Porter ME, Goldman KJ, Serra D. Age and growth of sharks: do vertebral band pairs record age? Mar Freshw Res. 2018;69: 1440-1452. doi: 10.1071/MF17279

69. Natanson LJ, Deacy BM. Using oxytetracycline validation for confirmation of changes in vertebral band-pair deposition rates with ontogeny in sandbar sharks (*Carcharhinus plumbeus*) in the western North Atlantic Ocean. Fish bull. 2019;117: 50-58. doi: 10.7755/FB.117.1.6

70. Casey JG, Pratt Jr. HL, Stillwell CE. Age and growth of the sandbar shark (*Carcharhinus plumbeus*) from the western North Atlantic. Can J Fish Aquat Sci. 1985;42: 963-975.

71. Francis MP, Campana SE, Jones CM. Age under-estimation in New Zealand porbeagle sharks (*Lamna nasus*): is there an upper limit to ages that can be determined from shark vertebrae? Mar Freshw Res. 2007;58: 10-23.

72. Passerotti MS, Andrews AH, Carlson JK, Wintner SP, Goldman KJ, Natanson LJ. Maximum age and missing time in the vertebrae of sand tiger shark (*Carcharias taurus*): validated lifespan from bomb radiocarbon dating in the western North Atlantic and southwestern Indian Oceans. Mar Freshw Res. 2014;65: 674-687. doi: 10.1071/MF13214

73. Hamady LL, Natanson LJ, Skomal GB, Thorrold SR. Vertebral bomb radiocarbon suggests extreme longevity in white sharks. PLoS ONE. 2014;9: e84006. doi: 10.1371/journal.pone.0084006

74. Natanson LJ, Skomal GB. Age and growth of the white shark, *Carcharodon carcharias*, in the western North Atlantic Ocean. Mar Freshw Res. 2015;66: 387-398. doi: 10.1071/MF14127

75. Ogle DH. Introductory Fisheries Analyses with R. Boca Raton: CRC Press; 2016.

76. Pimiento C, Cantalapiedra JL, Shimada K, Field DJ, Smaers JB. Evolutionary pathways toward gigantism in sharks and rays. Evolution. 2019;73: 588-599. doi: 10.1111/evo.13680

77. Parker HW, Stott FC. Age, size and vertebral calcification in the basking shark, *Cetorhinus maximus* (Gunnerus). Zool Meded. 1965;40: 305-314.

78. Peters RH. The ecological implications of body size. New York: Cambridge University Press; 1983.

79. Makarieva AM, Gorshkov VG, Li B-L. Why do population density and inverse home range scale differently with body size? Implications for ecosystem stability. Ecol Complex. 2005; 2: 259-271.

80. Ohlberger J. Climate warming and ectotherm body size – from individual physiology to community ecology. Funct Ecol. 2013;27: 991-1001. doi: 10.1111/1365-2435.12098

81. Vermeij GJ. Gigantism and its implications for the history of life. PLoS ONE. 2016;11: e0146092. doi:10.1371/journal.pone.0146092

82. García VB, Lucifora LO, Myers RA. The importance of habitat and life history to extinction risk in sharks, skates, rays and chimeras. Proc R Soc B. 2008;275: 83-89.

Supporting Information

S1 Fig. Placoid scales found associated with vertebrae of EMRG-Chond-SK-1. (A) type 1, “six-keeled scales”; (B) type 2, “three-keeled scales”; (C) type 3, “knob-like scales”.

Figure Click here to access/download;Figure;Figure 1.tif

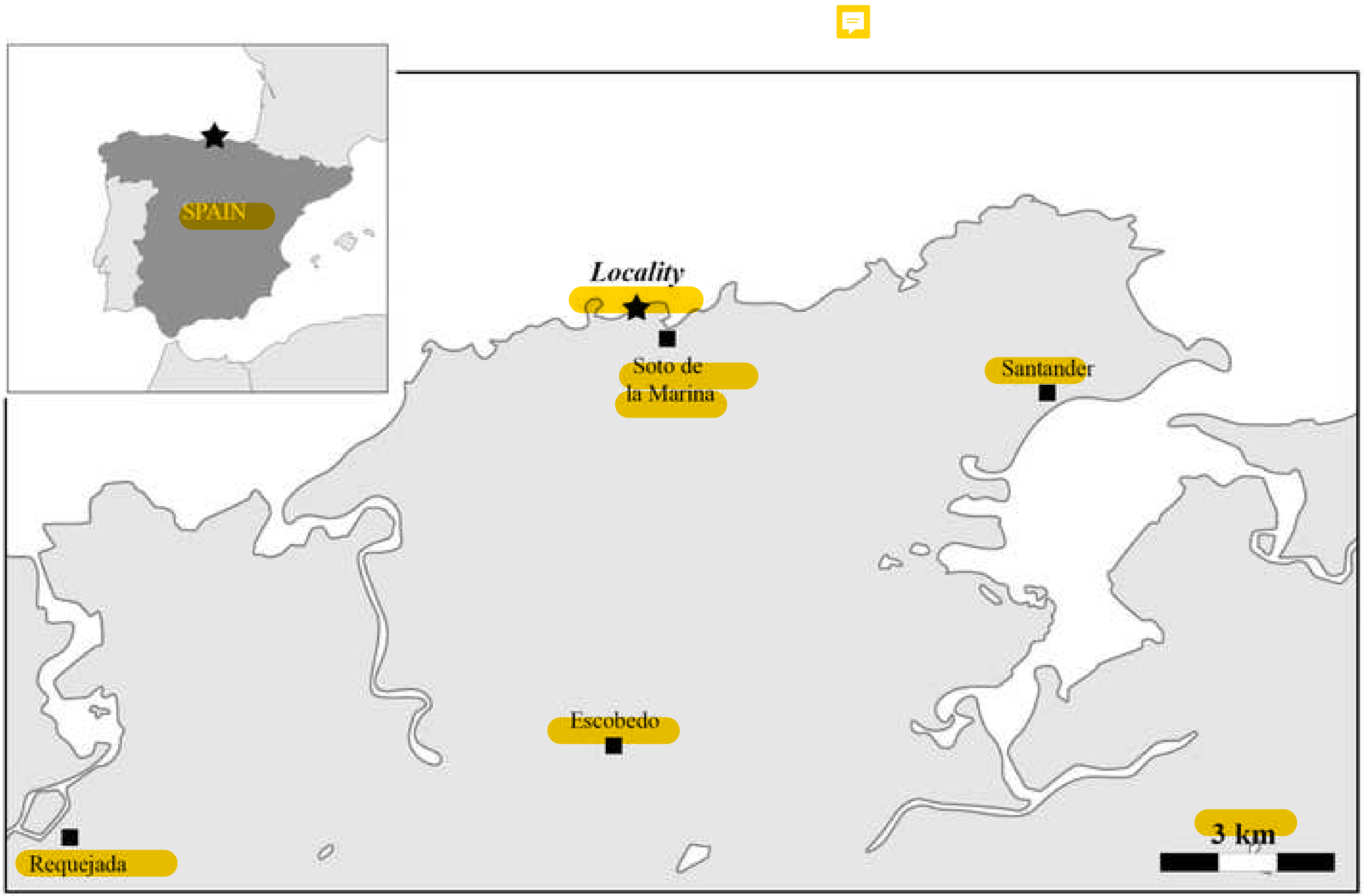

Click here to access/download;Figure;Figure 2.tif

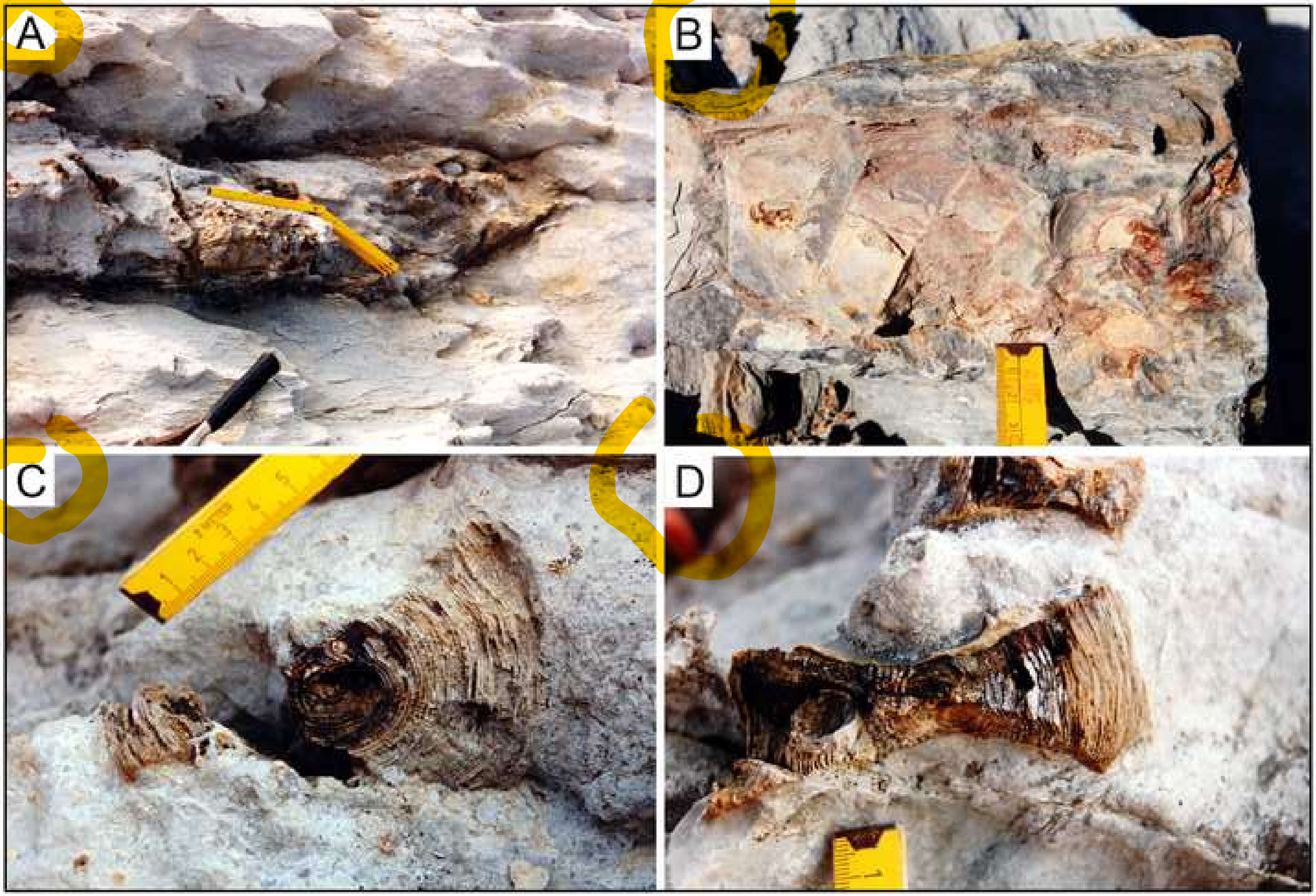

Figure
Click here to access/download;Figure;Figure 3 (1).tif

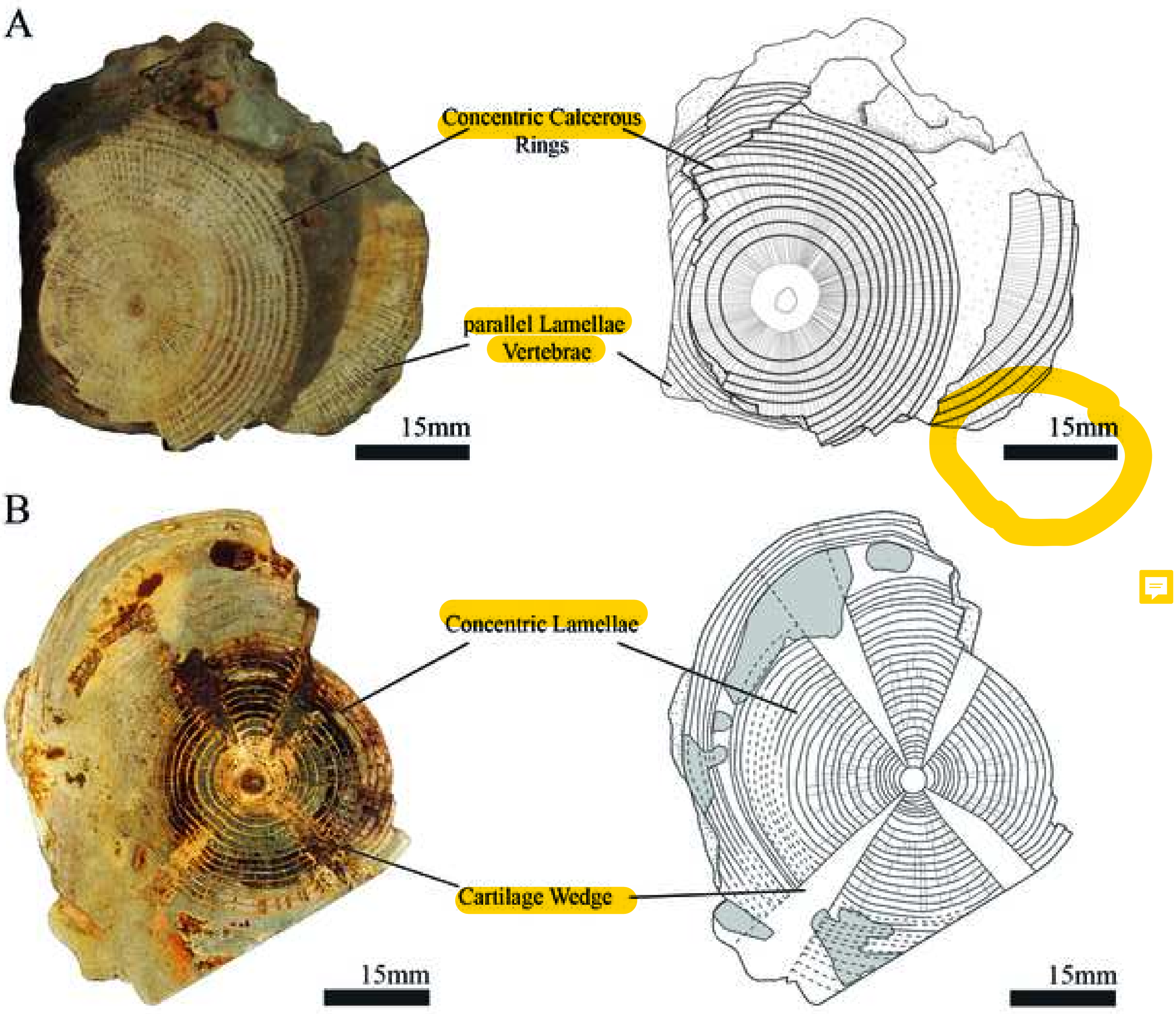

Figure
Click here to access/download;Figure;Figure 4.tif

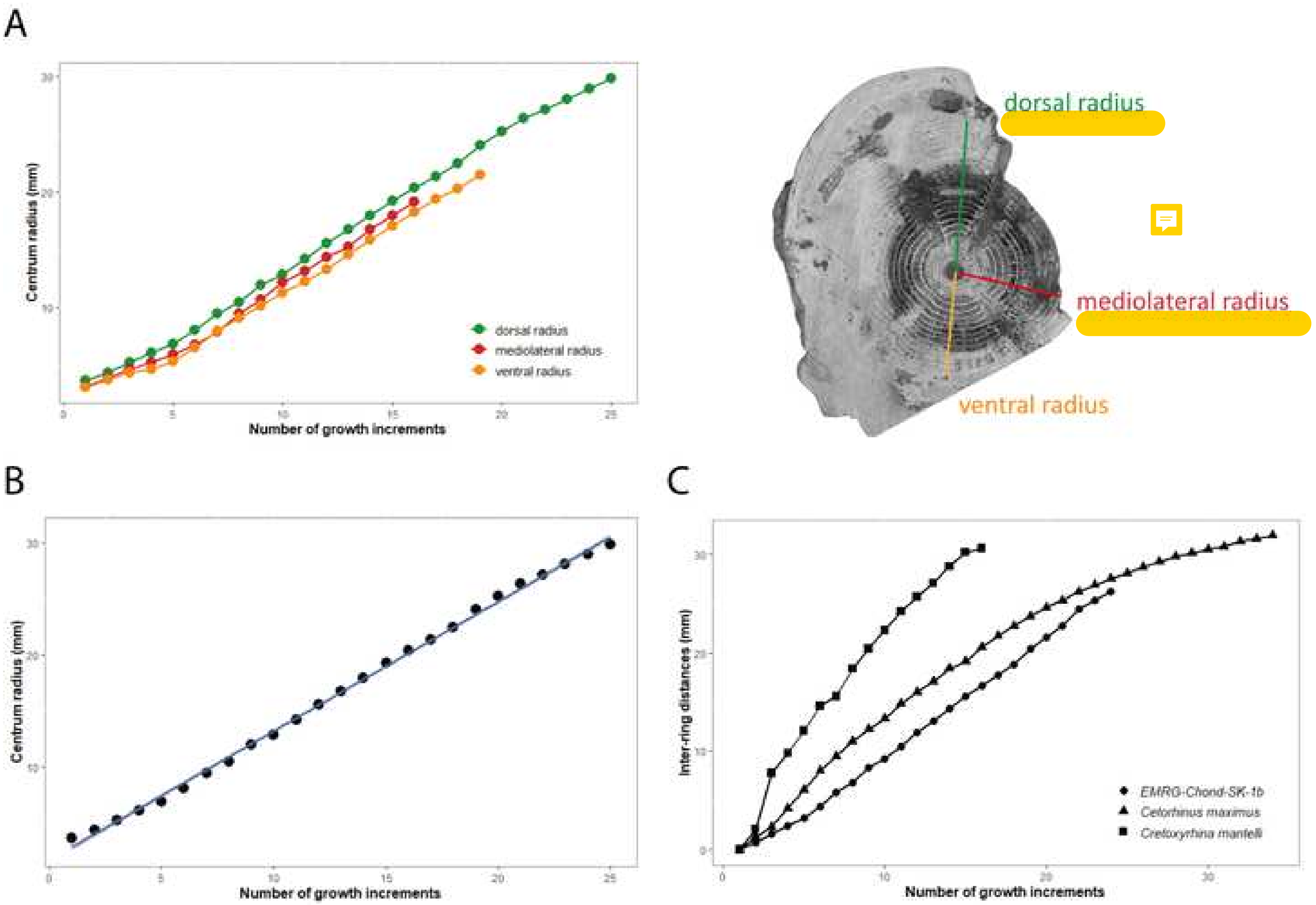

Click here to access/download
Supporting Information
Fig_S1 (2).tif
